# THE DEVELOPMENTAL WINDOWS WHEN EXPOSURE TO ANTIBIOTICS INCREASES THE RISK OF EARLY ONSET COLORECTAL CANCER

**DOI:** 10.1093/geroni/igad104.3132

**Published:** 2023-12-21

**Authors:** Nichelle Tomlin, Niti Jani, Marie Pena

**Affiliations:** Claflin University, Summerville, South Carolina, United States; Columbia, South Carolina, United States; University of South Carolina, Columbia, South Carolina, United States

## Abstract

Colorectal cancer is the third most diagnosed cancer and the second leading cause of cancer deaths in the US. About 50,000 people die from colorectal cancer each year. Early onset Colorectal cancer (EOCRC) is a colon and rectum cancer occurring in people under 50. There has been a decline in cases in people over 50 due to healthier lifestyle choices. However, in the last few decades there has been a steady increase in EOCRC cases, and we do not know why. The increase in EOCRC has paralleled the increased use of antibiotics. Antibiotics are especially important for fighting infections, but overuse has become an epidemic. Our study tested the hypothesis that exposure to antibiotics in early life causes dysbiosis and inflammation in the colon that can lead to an increased risk for EOCRC. To test the hypothesis a mice model was used. Female mice who have given birth are giving antibiotic treatment for 7 days, so the children are exposed to the antibiotics through their mother’s milk at an early age. The children are weaned from their mothers and at 5 weeks old they are given Azoyxmethane (AOM) treatment once a week for 6 weeks. Fecal pellets were collected before and after the AOM treatment of the kids and antibiotic treatment of the mother. The results show if given multiple dosages of amoxicillin there will be tumor burden in the proximal colon which can increase the risk for EOCRC.

